# Medico-Legal Analysis of General Surgery Cases in Greece: A 48 Year Study

**DOI:** 10.7759/cureus.16205

**Published:** 2021-07-06

**Authors:** Lambros Tzoumas, Evangelia Samara, Konstantinos Tzoumas, Petros Tzimas, Konstantinos Vlachos, Georgios Papadopoulos

**Affiliations:** 1 Department of Anesthesiology and Postoperative Intensive Care, Faculty of Medicine, School of Health Sciences, University Hospital of Ioannina, Ioannina, GRC; 2 Department of Anaesthesiology, Onassis Cardiac Surgery Center, Athens, GRC; 3 Department of Anesthesiology and Postoperative Intensive Care, Faculty of Medicine, School of Health Sciences, University of Ioannina, Ioannina, GRC; 4 Department of Surgery, Faculty of Medicine, School of Health Sciences, University of Ioannina, Ioannina, GRC

**Keywords:** surgery general, bariatric surgery, laparoscopic surgery, medical liability, medical malpractice, medical error

## Abstract

Introduction

Surgery can be dangerous not only for patients, but it also carries a risk for the surgeon in case of medical error, which can affect their future medical career. The purpose of our research is to assess the current situation regarding medical liability in General Surgery in Greece, the reasons for the allegations of medical malpractice as well as the relationship between these issues and the court results.

Methods

Published court decisions of criminal, civil, administrative and disciplinary content were searched in legal information banks, between year 1973 and 2020. Court decisions were analyzed by an expert, a specialist general surgeon and an anesthesiologist, for the causes of death and the correctness of the court decision in collaboration with the lawyers of the investigation.

Results

588 court decisions were retrieved, out if which 103 (17.751%) criminal (n = 81), or civil and administrative decisions (n ​​= 22) for surgeons. Out of a total of 81 first and second instance criminal cases and appellate court decisions of the Supreme Court, 27 cases concerned negligent homicide, 16 cases concerned negligent bodily harm and seven were acquittals. Out of 22 civil cases decisions, awarding or not awarding compensation, three cases concerned negligent homicide, seven bodily harm and two were acquittals. 11 cases of negligent homicide concerned laparoscopic and bariatric surgical procedures.

Conclusions

Laparoscopic surgery represents one of the most important surgical developments in the last 30 years. However, they represent a great proportion of the cases concerning medical malpractice in the greek legal system. It is important to have a national center for reporting misdiagnosis and complications and a medical liability system that will facilitate improved diagnosis, learning from diagnostic errors and delays in diagnosis, in order to avoid similar cases of malpractice in the future.

## Introduction

Surgery can be dangerous not only for patients, but it also carries a risk for the surgeon in case of medical error [1-3]. In a study by Mark et al., the overall complication rate in patients undergoing general surgery procedures was 30.3% (413 patients). The major complication rate was 16.2%, and the percentage of serious complications that could have been avoided was 53.4%. Mistakes in patient care contributed to 38 (30%) of 128 deaths [4].

In Moreira's study, alleged cases of medical liability in general surgery accounted for 11.2% of the total cases analyzed [5]. Medical liability delimitation and evaluation is a major judicial issue, as it often determines a doctor’s future medical career. In case of malpractice, it is checked whether the medical behavior as an action followed the rules governing medical ethics. This includes the patient’s informed consent and the control of whether the medical act was performed according to the rules of medical art and science. Rules are determined for each medical specialty by the respective scientific companies as guidelines. Medical actions are judged as opposed to the expected actions by the average prudent specialist doctor [6].

Analysis of medical malpractice court decisions concerning surgeons can provide a detailed picture of the causes of patient mortality and morbidity. The purpose of our research is to assess the current situation regarding medical liability in General Surgery in Greece, in order to better understand the reasons for the allegations of medical malpractice as well as the relationship between these issues and the court results, as an initial step to future more prudent management of similar cases.

## Materials and methods

Published court decisions of criminal, civil, administrative, and disciplinary content, between year 1973 and 2020 were searched in the legal information banks Nomos, Sakkoulas online.gr, Bank of the Athens Bar Association as well as in legal magazines, such as Nomiko Vima, Hellenic Justice, Criminal Chronicles, Criminal Justice.

Age of the patients, sex, date of operation, causes that led to the adverse outcome, and duration of the judicial recording were recorded. The court decisions were analyzed by an expert, a specialist general surgeon, and an anesthesiologist, for the causes of death and the correctness of the court decision in collaboration with the lawyers of the investigation.

## Results

A total of 588 court decisions were retrieved, of which 103 (17.5%) concerned criminal (n = 81), or civil and administrative (n ​​= 22) decisions for surgeons. Among the criminal cases, out of a total of 81, first and second instance and appellate court decisions of the Supreme Court, 27 cases concerned negligent homicide, 16 cases concerned negligent bodily harm, and seven were acquittals. Among civil cases awarding or not awarding compensation, out of a total of 22 decisions, three cases concerned negligent homicide, seven bodily harm, and two were acquittals.

Table 1 shows the patient's sex, age, risk classification according to the American Society of Anesthesiologists (ASA), and the duration of the litigation.

**Table 1:** Biometric characteristics of patients involved and duration of the legal dispute (N = 58). ASA: American Society of Anesthesiologists

Men/Women	35/23
Mean age (years)	42 ±22,8 (0,6 – 79)
ASA I, II/III	40/18
Legal dispute duration (years)	8,08± 2,41 (2 –13)

Table 2 shows the causes of negligent death. In four cases five trainees were convicted, while in one case, both the consultant and the trainee were convicted.

**Table 2:** Causes of negligent homicide. Criminal and civil cases (n=29)

Criminal cases-Negligent homicide	12
Laparoscopic surgery- Hemorrhage	5
Laparoscopic surgery- Peritonitis	3
Bariatric surgery- Peritonitis	3
Sigmoidectomy- Peritonitis	1
Missed diagnosis	7
Missed or untimely diagnosis of peritonitis	5
Missed thoracic aneurysm rupture diagnosis	1
Missed abdominal aortic aneurysm rupture diagnosis	1
Airway	3
Liposuction, sedation-airway obstruction	1
Difficult intubation, carotid and jugular vein erosion.	1
Sedation in a subdural hematoma patient	1
Other causes	7
Snakebite, hemodialysis was not offered	1
Single ectopic kidney removal considered as malignant tumor	1
Post total thyroidectomy mediastinitis	1
Bowel obstruction, delayed surgery	1
Esophagus surgery-gastric tube removal-no imaging performed	1
Cliff fall, rhabdomyolysis syndrome	1
Surgeon and Department conviction	1
Strangulated inguinal hernia - obstructive ileus	1

In two cases of negligent homicide, the anesthesiologist was also convicted. These included a case of carotid-pharyngeal erosion during emergency tracheotomy due to difficult intubation and a case of airway obstruction during procedural sedation, as the patient was not monitored. In one of the cases of negligent homicide, the internist and cardiologist were also convicted for wrong diagnosis of a thoracic aneurysm rupture. In another case, an internist was convicted as well for misdiagnosis of a ruptured abdominal aortic aneurysm.

Informed consent was incomplete in four cases. In one case of negligent homicide, the physician was additionally convicted for passive bribery. 37.9% of negligent homicides (11 cases) concerned laparoscopic and bariatric surgical procedures. The cause of death in these cases was vascular or intestinal erosion during the trocar entrance, while there was a delayed diagnosis and management of the lesion. In 24% of cases, the conviction was based on wrong or untimely diagnosis.

Finally, Table 3 shows the causes of bodily harm caused by surgical negligence in criminal and civil cases in which compensation was awarded.

**Table 3:** Causes of bodily harm due to surgical negligence, in criminal and civil cases awarding compensation for mental pain or moral damage

Criminal bodily harm cases	
Bariatric surgery	2
Laparoscopy, aortic rupture	2
Other causes	
Cholecystectomy, re-operation without drainage placement	1
Harm due to inadequate hospital equipment maintenance	1
No chest CT ordered in a traffic accident patient	1
Postoperative foot ulcers – inappropriate treatment	1
Inguinal hernia repairment – ilioinguinal neurotmesis	1
Missing informed consent- inguinal hernia procedure, arterial thrombosis	1
Upper abdomen tumor operation, while chemotherapy was indicated	1
Civil or administrative cases rewarding compensation	
Sigmoidectomy - ureteral suture entrapment, kidney necrosis.	2
Unsuccessful adrenal tumor removal during the initial operation.	1
Mesh inflammation post hernia repairment (also clinic’s liability)	1
Improper wound care	1
Incorrect single ectopic kidney removal	1
Limb ischemia during inguinal hernia repairment, heparin not administered	1

## Discussion

In our study, the most common cause of death (37.9%) was laparoscopy complications in cholecystectomy and bariatric procedures, due to delayed management of vascular or intestinal injury. The average duration of litigation was eight (two to 13) years and the average compensation was 115,190 euros. In contrast to the international literature in our investigation there were only complaints of death and serious bodily harm, while the duration of the litigation was much longer and the compensation significantly lower.

On the contrary, in de Reuve' s study, the dispute over poor medical practice after laparoscopic surgery was much shorter, with a median duration of two years, while responsibility for malpractice was identified in 16 (18%) of 88 cases. [7]. Kienzle reported malpractice in 25 of 44 complaints of laparoscopic cholecystectomy procedures, mainly concerning bile duct injuries. In one case there was insufficiently informed consent, an element also mandatory by the Greek law [8].

In the study by Wind et al., 18% of all laparoscopic complications were associated with trocar entry. Out of 51 structure injuries, 18 concerned vascular structures during exclusively closed entrance techniques. Only 19 of the cases were detected intraoperatively. There was no mortality and the main reason for patients to have claimed compensation was prolonged hospitalization and related costs [9]. On the contrary, in Moreira's research, the majority of complaints (75.4%) in general surgery procedures, mainly laparoscopic cholecystectomy, concerned patients’ deaths [5].

In our research, the wrong or untimely diagnosis, mainly for intestinal inflammations with consequent peritonitis, had an incidence of 24%. In Jung' s study, misdiagnosis (30.8%) was the most common cause of complaints, followed by post-operative care (27.7%). The duration of the legal dispute was still shorter, with a mean of 3.2 years. Breach of duty while on medical care was reported in 49 cases, breach of informed consent in seven cases, while violation of both in five cases [10].

Choudhry et al. reported that failure to diagnose and manage intestinal obstruction in a timely manner accounted for 69% of cases. Mortality occurred in 61% of cases, while 54% of the cases were finally acquittal for the accused physician. The average compensation was $1,136,220 [11].

In the research of Brown et al., malpractice cases included diagnostic errors (37%) and poor execution of the indicated procedure (17%). Emergency physicians were the primary specialty involved. The most common health conditions to lead to claims were cardiovascular incidents, fractures and acute abdomen. 29% of claims for compensation were settled, while 70% received no compensation. According to the study, average claims and court costs overdoubled with time, while claim ceases as well as claims awarded payment decreased [12]. On the other hand, Weber et al. reported that the percentage of claims for malpractice in bariatric surgery decreased, while the average compensation increased within a period of 20 years [13].

In our study, cases of bariatric surgery represented 18.5% of the total cases. Delayed diagnosis and complication management was the main error found. Bariatric surgery for morbid obesity has become an effective and acceptable treatment for prolonged weight loss. However, it carries significant risks and continues to significantly affect general surgery practice.

In Radwan' s study, 81 (19%) out of 426 claims concerning visceral and digestive surgery were related to bariatric surgery. Fistula was the most common complication recorded (43.67%). The duration of the dispute was on average two (one to six) years [14]. Regarding bariatric surgery, in a paper including 59 bariatric surgery procedures that led to the complaints, 40% concerned laparoscopic adjustable gastric band, 28% gastric bypass surgery and 23% sleeve gastrectomy. Immediate postoperative complications included technical related surgical complications and infections, leading to reoperation for 78% of the patients. Negligence, mainly delay, was detected in one third of the cases [15].

To improve misdiagnosis or late diagnosis, both in laparoscopic procedures and general surgery in general, there should be no underemployment with the patient and laboratory examinations, and teamwork and approaches to spot deficiencies in clinical practice should be enhanced. It is important to have a national center for reporting misdiagnosis and complications and a medical liability system that will facilitate improved diagnosis, learning from diagnostic errors and delays in diagnosis [16].

In case of malpractice, in order to improve the legal opinion of the defendant doctor, which is known to be insufficient, a clear, extensive and detailed documentation of the medical examination findings and the planned operation indications is required, along with the patient’s informed consent. Informed consent is mandatory in greek medical practice and violation has led to physician convictions [17]. Extensive, detailed, careful and responsible operation reporting is also required, as well as a systematic, orderly and well-designed management of postoperative complications to address the accusation of malpractice. The building of mutual trust between surgeons and lawyers is protected by a comprehensive documentation and enables a clear description and formulation of the medical report [18].

## Conclusions

Laparoscopic surgery represents one of the most important surgical developments in the last 30 years. It has significant advantages over laparotomy. However, its complications vary between hospitals and surgeons. Surgical skills and experience of surgeons seem to play an important role. To improve its results continuous training in technique and crisis management in case of serious complications is required. In our study, laparoscopic surgery occupied a great proportion of malpractice allegations.

To avoid misdiagnosis or delay diagnosis in general surgery, constant employment with the patient is required. In case of malpractice, a detailed documentation of the medical examination findings and the planned operation indications is mandatory, along with the patient’s informed consent. Clear documentation facilitates the trust between the medical and the legal representatives, as well as their study for future reference, in order to avoid repeating similar errors in the future.
